# Rapidly Growing Nontuberculous *Mycobacterium* Wound Infections Among Medical Tourists Undergoing Cosmetic Surgeries in the Dominican Republic — Multiple States, March 2013–February 2014

**Published:** 2014-03-07

**Authors:** David Schnabel, Joanna Gaines, Duc B. Nguyen, Douglas H. Esposito, Alison Ridpath, Kari Yacisin, Jose A. Poy, Jocelyn Mullins, Rachel Burns, Virginia Lijewski, Nora P. McElroy, Nina Ahmad, Cassandra Harrison, Ellen J. Parinelli, Amanda L. Beaudoin, Leah Posivak-Khouly, P. Scott Pritchard, Bette J. Jensen, Nadege C. Toney, Heather A. Moulton-Meissner, Edith N. Nyangoma, M. Anita Barry, Katherine A. Feldman, David Blythe, Joseph F. Perz, Oliver W. Morgan, Phyllis Kozarsky, Gary W. Brunette, Mark Sotir

**Affiliations:** 1 Maryland Department of Health and Mental Hygiene; 2 Division for Global Migration and Quarantine, National Center for Emerging and Zoonotic Infectious Diseases, CDC; 3 Division of Healthcare Quality and Promotion, National Center for Emerging and Zoonotic Infectious Diseases, CDC; 4 New York City Department of Health and Mental Hygiene, New York, New York; 5 Connecticut Department of Public Health; 6 Massachusetts Department of Public Health; 7 New York State Department of Health; 8 New York State Metropolitan Area Regional Office; 9 New York State Orange County Health Department; 10 Pennsylvania Department of Health; 11 Montgomery County Health Department, Norristown, Pennsylvania; 12 Florida Department of Health; 13 Boston Public Health Commission, Boston, Massachusetts; 14 Dominican Republic Country Office, Center for Global Health, CDC

In August 2013, the Maryland Department of Health and Mental Hygiene (MDHMH) was notified of two persons with rapidly growing nontuberculous mycobacterial (RG-NTM) surgical-site infections. Both patients had undergone surgical procedures as medical tourists at the same private surgical clinic (clinic A) in the Dominican Republic the previous month. Within 7 days of returning to the United States, both sought care for symptoms that included surgical wound abscesses, clear fluid drainage, pain, and fever. Initial antibiotic therapy was ineffective. Material collected from both patients’ wounds grew *Mycobacterium abscessus* exhibiting a high degree of antibiotic resistance characteristic of this organism (1).

Attempting to identify additional cases, MDHMH posted Epi-X* alerts in August, November, and December 2013. Health department officials in Connecticut, Florida, Massachusetts, New Jersey, New York, Pennsylvania, Boston, and New York City, and CDC officials joined MDHMH to investigate possible cases reported. Official health alerts from state and local health departments and notifications through the Emerging Infections Network and the American Society of Plastic Surgeons requested that health-care providers and the public health community report additional patients. A probable case was defined as a soft-tissue infection unresponsive to standard antibiotic therapy in a patient who had undergone cosmetic surgery in the Dominican Republic after March 1, 2013. A confirmed case was defined as a probable case testing positive for RG-NTM. Patients with probable and confirmed infection were interviewed by using a standardized questionnaire; a systematic abstraction of patients’ medical records is ongoing. Pulsed-field gel electrophoresis of available isolates from patients associated with clinic A is being performed at CDC and the New York City Department of Health and Mental Hygiene.

As of February 21, 2014, a total of 19 cases were identified from five states (New York, 11; Massachusetts, three; Connecticut, two; Maryland, two; and Pennsylvania, one). Sixteen (84%) cases were confirmed, and three (16%) were probable. All patients are female (aged 18–59 years). Twelve (63%) reported undergoing surgery at clinic A, and seven (37%) reported surgery at seven other Dominican Republic surgical clinics. The most common cosmetic surgical procedures were liposuction (74%), abdominoplasty (58%), and breast implantation (32%); all procedures occurred during March–November 2013 (Figure), and illness onsets occurred during April–November 2013. Fourteen (74%) were hospitalized in the United States and required multiple therapeutic and corrective surgical procedures and long courses of antibiotics; five were treated as outpatients. No deaths were reported. Of the 16 confirmed cases, 13 (81%) were *Mycobacterium abscessus* infections; two (12%) were *M. fortuitum* infections; and one (6%) is pending final speciation. Of the 18 patients who were interviewed, 13 (72%) were born in the Dominican Republic.

CDC notified Dominican public health authorities of the outbreak investigation and recommended patient follow-up and onsite assessment of infection control practices at the implicated clinics. Clinic A has been closed temporarily by Dominican authorities. This and other outbreaks underscore the risk for infection, including RG-NTM infection, resulting from medical tourism (2,3). CDC advises all persons planning to receive surgical care outside the United States to verify that the health-care provider and facility they are considering using are licensed and accredited by an internationally recognized accreditation organization before proceeding (4,5). These findings indicate that health-care providers consider RG-NTM among patients with a history of cosmetic surgery in the Dominican Republic who also have a surgical-site infection that fails to respond to standard therapy.

## Figures and Tables

**FIGURE:**
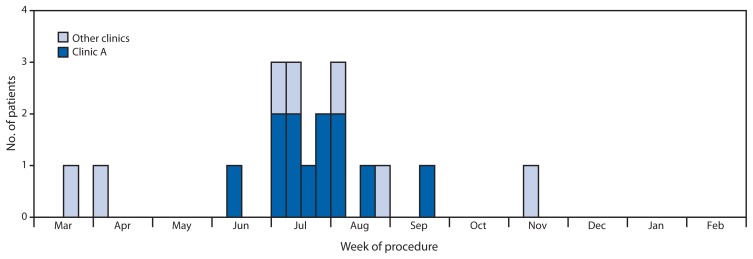
Number of U.S. patients (N = 19) with rapidly growing nontuberculous *Mycobacterium* infections associated with cosmetic surgery in the Dominican Republic, by week of procedure — March 2013–February 2014

## Footnotes

* Epi-X is a CDC-operated, web-based information exchange for public health practitioners. Additional information available at http://www.cdc.gov/epix.
